# Exercise combined with artificial gravity and exercise only countermeasures prevent organ and blood vessel morpholgical changes induced by 55 days HDT bedrest

**DOI:** 10.3389/fphys.2024.1482860

**Published:** 2024-11-28

**Authors:** P. Arbeille, K. Zuj, L. Guillon

**Affiliations:** UMPS-CERCOM (Unit Med Physiol Spatiale) Faculte de Medicine Universite de Tours, Tours, France

**Keywords:** bedrest, echography, organ and vessel imaging, countermeasure, exercise

## Abstract

**Background:**

Changes in blood vessel properties have been identified with confinement, spaceflight, bedrest, and dry immersion. Subsequently, it was suspected that other organs may also be affected in these extreme environments. The purposes of the current study were to determine the effects of head-down bedrest (HDT) on cardiovascular and organ measurements made using ultrasound imaging similar to that currently available on the International Space Station, and to evaluate the efficacy of two different countermeasure protocols in preventing any observed changes in the ultrasound measurements with HDT.

**Methods:**

Ultrasound measures were conducted on 24 individuals (3 groups of 8) pre HDT and on day 55 of the HDT. The control group (C°) remained in passive HDT for the 55 days, the C1 group performed aerobic exercise daily (EX), and the C2 group practiced aerobic exercise under artificial gravity conditions (EX-AG). Fifteen parameters were measured on 10 different organs and blood vessels including the right common carotid artery, abdominal aorta, right tibial artery, left ventricle, right jugular vein, portal vein, right kidney, cervical and lumbar vertebra, and the vastus intermedius muscle.

**Results:**

HDT resulted in changes for many of the parameters investigated. Observed changes in carotid IMT and distensibility, cardiac ejection fraction, portal vein diameter, and vastus intermedius muscle thickness were attenuated with EX and EX-AG, with EX-AG having a greater effect than exercise alone on measures of carotid distensibility.

**Conclusion:**

Results from this study indicate changes in many structures assessed with ultrasound imaging after 55 days of HDT bedrest with some changes being attenuated with the two investigated countermeasure protocols.

## Introduction

Cardiovascular adaptations to real and simulated microgravity have been well documented. Common carotid artery wall thickness has been found to increase with reductions in distensibility after 6 months spaceflight, prolonged confinement, and dry immersion in approximately 75% of the subjects (Arbeille et al., 2001; Arbeille et al., 2014; Arbeille et al., 2015; Hughson et al., 2016; Arbeille et al., 2017a; Yuan et al., 2019). These changes have been associated with alterations in common carotid artery wall properties evaluated through the processing of the ultrasound frequency signal (Arbeille et al., 2021b). Additionally, jugular vein and portal vein size have also been found to increase with similar exposure to real and simulated microgravity (Arbeille et al., 2001; Arbeille et al., 2014; Marshall-Goebel et al., 2018; Marshall-Goebel et al., 2019; Arbeille et al., 2021a; Patterson et al., 2023). While it is believed that the cardiovascular adaptations primarily result from the fluid shifts associated with microgravity exposure, other factors, such as induced confinement physical and mental stress, or absence of physical activity may also contribute to the observed adaptations.

Physiological changes have also been noted in other anatomical regions. Ultrasound assessments during spaceflight have revealed abnormal vertebral disc shape (herniation) and muscle atrophy with these changing being confirmed postflight using MRI (Belavy et al., 2016; Bailey et al., 2017; Garcia et al., 2017; Harrison et al., 2018). However, limited work has been done to investigate potential changes in organ structure with real and simulated microgravity exposure. As ultrasound is currently the only diagnostic imaging modality aboard the International Space Station (ISS), the current study used ultrasound to investigate vascular and organ structures suspected to be affected by bedrest in alignment with a similar series of ultrasound assessments currently used on the ISS (CIPHER).

The current study was designed to investigate both the effects of 55 days of 6° head-down tilt bedrest (HDT) on blood vessel and organ structures and to evaluate the efficacy of two countermeasure protocols, aerobic exercise alone (EX), and aerobic exercise combined with artificial gravity exposure (EX-AG), in reducing or preventing any inappropriate changes induced by HDT. It was hypothesized that 55 days of HDT would affect most of the blood vessels and organs investigated. It was also hypothesized that both countermeasures would attenuate the effects of bedrest and that exercise with artificial gravity exposure would show a greater protective effect than exercise alone.

## Research design and methods

### Subjects

Data were collected from 24 males who participated in the 6° HDT bedrest “BRACE” experiment. Participants had a mean age of 29.4 ± 5.6 years, height of 176 ± 6.4 cm, and body mass index of 23.88 ± 1.83 kg/cm^2^. All study protocols and procedures were in accordance with the Declaration of Helsinki and were approved by the local research ethics committee, CPP Ile de France VI on 12/19/2022. Decision Favorable- Authorization ANSM on 11/01/2023 - N° ID-RCB: 2022-A02074-39. Each participant gave written informed consent before participating in the study.

### Experimental protocol

Participants were assigned to one of three experimental conditions before completing 55 days of continuous, 6° HDT bedrest. The control group (C°; n = 8) completed the bedrest without any countermeasures. Participants in the exercise countermeasure group (EX; C1; n = 8) used a supine cycle ergometer to perform daily aerobic exercise during HDT. The program was similar to that used on the ISS where participants started at 40% of VO_2_max for 5 minutes followed by 2-min intervals at 65%, 70%, 80%, 70%, and 65% VO_2_max each separated by 2 minutes of cycling at 40% VO_2_max and ended with 3 min of cycling at 40% VO_2_max.

The third group (EX-AG; C2, n = 8) completed aerobic exercise with artificial gravity exposure. Artificial gravity (AG) was generated with a 2.8 m radius human centrifuge where participants remained supine with their head towards the centre of the centrifuge. The applied level of AG was determined for each participant based on their respective cardiovascular orthostatic threshold (OT) and the AG level for presyncope (PS). Threshold values were determined at the start of the study using an AG tolerance centrifuge run. This run started with 10 min of centrifugation at 0.6 Gz acceleration (at heart level) followed by 0.1 Gz increases every 3 minutes until participants experienced presyncopal symptoms or monitoring of blood pressure, heart rate, and cardiac output showed marked reductions indicating the onset of presyncope.

During the bed rests period, C2 participants (EX-AG) completed the same cycling protocol as the C1 group (EX), but with the application of AG. Participants started peddling with AG applied at 0.15 Gz less than the individual’s OT threshold with AG increased by 0.15 Gz every 4 minutes, synchronized with the cycling intervals. AG was only increased up to 70% of the participants tolerance level (0.7(PS - OT) + OT) and was decreased by 0.15 Gz every 4 minutes until the cycling protocol was completed.

### Measurements

Ultrasound assessments were performed during the 1-week ambulatory period before the start of HDT bedrest and on day 55 of the bedrest. The device used was the CNES Sonoscanner echograph (Paris, France) equipped with motorized probes (superficial 17 MHz, abdominal 3.5 MHz, cardiac/transcranial 1.5 MHz) for 2D echography, volume acquisition, 3D reconstruction, RF modality (radio frequency), and speckle tracking post processing (Arbeille et al., 2018). This device is the ground model of the echograph presently in use onboard the ISS.

The following 10 organs and blood vessels were investigated: right carotid artery, right jugular vein, tibial artery, left ventricle, portal vein, aorta, cervical and lumbar vertebrae, right kidney, and the right vastus intermedius thigh muscle. For these 10 structures, 15 parameters were determined: carotid intima media thickness [CC IMT (mm)], carotid distensibility index [CC DI = (systolic diameter–diastolic diameter)/diastolic diameter], carotid blood flow (Qcc = vessel cross sectional area * mean blood velocity; ml/min), carotid vascular resistance index [CC RI = (systolic velocity–diastolic velocity)/systolic velocity], tibial artery vascular resistance index (RI tib = Negative end systolic velocity/peak systolic velocity), jugular vein volume [JV vol (cm^3^)], portal vein diameter [PV (mm)], aorta diam [Ao (mm)], left ventricle stoke volume [SV (cm^3^)], ejection fraction [EF = (systolic volume–diastolic volume)/diastolic volume], left ventricle myocardium posterior wall thickness [PW (mm)], anterior cervical intervertebral distance [C Vert (mm)], anterior lumbar intervertebral distance [L Vert (mm)], right kidney area [Kid A = long axis distance × short axis distance (mm^2^)], and vastus Intermedius thigh muscle thickness [VI thick (mm)]. These measurements were chosen as they have previously been investigated during the Deep Time experiment (Arbeille et al., 2023), dry immersion (Greaves et al., 2021), and the current NASA CIPHER routine ultrasound program. In addition to these measurements, all organs and vessels assessed were examined qualitatively for detection of possible structural abnormalities.

Ultrasound assessments were performed during the 1-week ambulatory period before participants started 60 days of HDT bedrest and at day 55 of the HDT. All ultrasound assessments were performed by two trained sonographers with the participant in a relaxed, supine position. The sonographer performed 2-dimensional imaging, arterial Doppler, and cardiac time motion as well as volume captures with 3D reconstructions and RF processing of each organ. The 3D modality allowed for post analysis of the ultrasound assessments and the optimization of the 2D images for accurate parameter measurements.

### Statistical analysis

To examine the effects of both exposure to HDT and differences in potential responses with the two countermeasure conditions, a two-way repeated measures analysis of variance was used with the main effects of HDT and experimental group (SigmaPlot 12.5, Systat Software Inc., San Jose, CA). The statistical software performed tests for both normality (Shaprio-Wilk test) and equal variance (Levene Median test) with all assessed variables passing both tests before further assessment. In the case of significant main effects or a significant interaction between HDT and experimental condition, Tukey *post hoc* testing was performed to determine significance of pairwise comparisons. Differences in responses to HDT between the control condition and the two countermeasure conditions were further assessed using a one-way analysis of variance to test for differences in the percent change with HDT. In the case of significant main effects, Tukey *post hoc* testing was again used to test all pairwise comparisons. For all tests, significance was set at p < 0.05 without correction for multiple comparisons. All data is reported as mean ± standard deviation.

## Results

**FIGURE 1:**
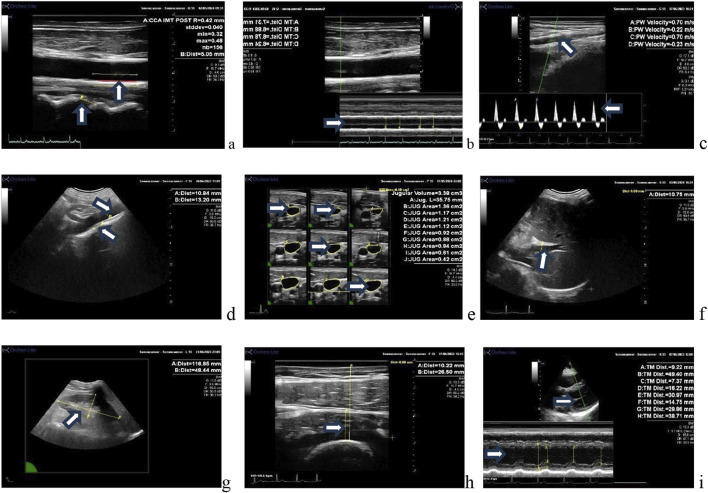
Ultrasound images of various vessels/organs assessed in this study. **(A)** Longitudinal view of the common carotid artery intima media thickness and cervical intervertebral distance, **(B)** carotid anterior and posterior wall time motion trace (systolic and diastolic diameter), **(C)** tibial artery image and Doppler velocity (lower limb vascular resistance). **(D)** Abdominal aorta diameter and lumbar intervertebral distance; **(E)** jugular vein volume (3D display); **(F)** portal vein diameter, **(G)** right kidney long axis area; **(H)** vastus intermedius thigh muscle thickness; **(I)** left ventricle parasternal long axis (volume, stroke volume posterior wall thickness).

**FIGURE 2:**
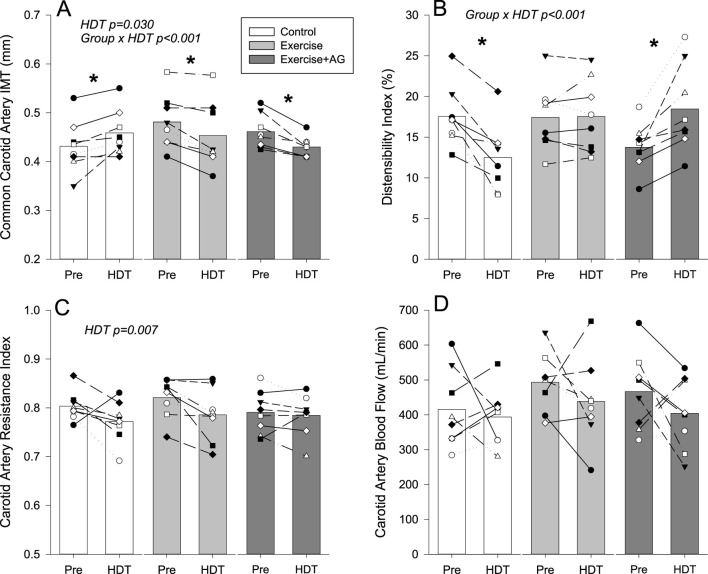
Mean (bars) and individual cardiovascular ultrasound measurements of common carotid artery IMT thickness **(A)**, distensibility index **(B)**, resistance index **(C)**, and quantitative blood flow **(D)** before (Pre) and after 55 days of HDT bedrest (HDT) for the control group (white bars), exercise countermeasure group (grey bars), and exercise with artificial gravity countermeasure group (dark grey bars). Significant main effects of HDT and group by HDT interactions are indicated. In the case of significant group by HDT interactions, groups that showed differences Pre to HDT are denoted by *p < 0.05.

**FIGURE 3:**
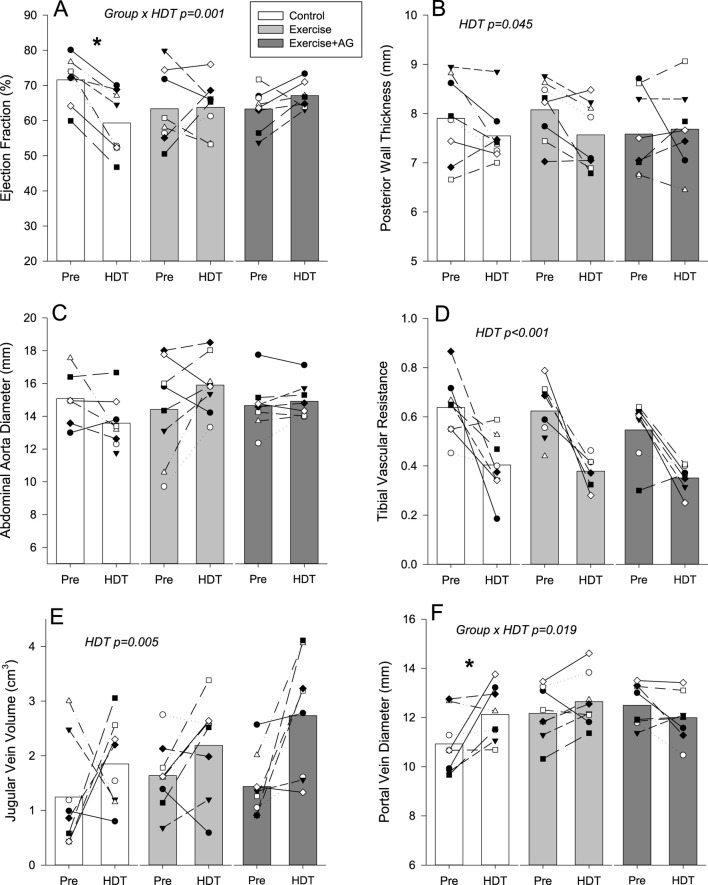
Mean (bars) and individual non-cardiovascular ultrasound measurements for cardiac ejection fraction **(A)**, posterior cardiac wall thickness **(B)**, abdominal aorta diameter **(C)**, tibial vascular resistance **(D)**, jugular vein volume **(E)**, and portal vein diameter **(F)** before (Pre) and after 55 days of HDT bedrest (HDT) for the control group (white bars), exercise countermeasure group (grey bars), and exercise with artificial gravity countermeasure group (dark grey bars). Significant main effects of HDT and group by HDT interactions are indicated. In the case of significant group by HDT interactions, groups that showed differences Pre to HDT are denoted by *p < 0.05.

**FIGURE 4:**
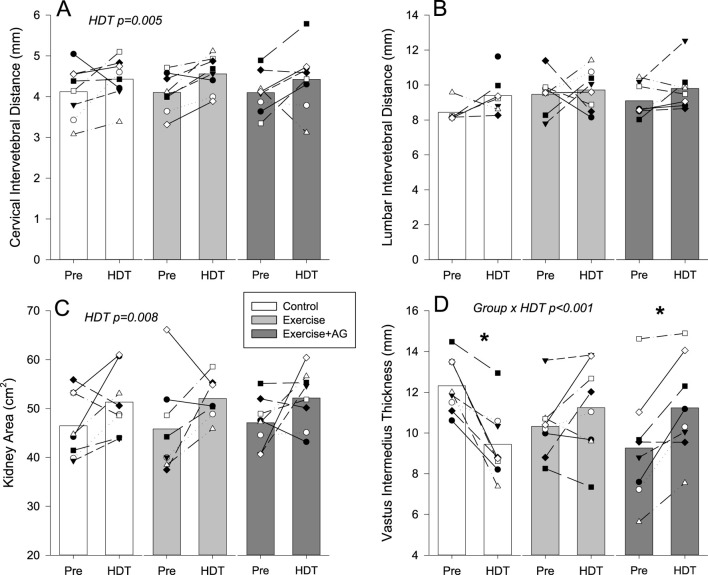
Mean (bars) and individual non-cardiovascular ultrasound measurements of cervical intervertebral distance **(A)**, lumbar intervertebral distance **(B)**, kidney area **(C)** and vastus intermedius thickness **(D)** before (Pre) and after 55 days of HDT bedrest (HDT) for the control group (white bars), exercise countermeasure group (grey bars), and exercise with artificial gravity countermeasure group (dark grey bars). Significant main effects of HDT and group by HDT interactions are indicated. In the case of significant group by HDT interactions, groups that showed differences Pre to HDT are denoted by *p < 0.05. End doc.

Ultrasound images for assessment were successfully obtained from each participant. Examples of 2D images used for assessment are presented in Figure 1. Qualitative evaluation of each of the structures investigated found no changes indicative of pathology development with 55 days of HDT bedrest. Quantitative measurements of the structures investigated with individual and mean responses are presented in Figures 2–4. Percent changes with HDT for each experimental condition and each variable assessed are presented in Table 1.

**TABLE 1:** Percent change in ultrasound measurements with HDT (mean ± SD) for carotid intima media thickness (CC IMT) and distensibility index (DI), cerebrovascular resistance (CC RI), carotid flow volume (Qcc), tibial vascular resistance (Tib RI), abdominal aorta diameter (Aorta), cardiac ejection fraction (EF), left ventricle myocardium posterior wall thickness (PW), jugular vein volume (JV vol), portal vein diameter (PV). Cervical (C Vert) and lumbar (L Vert) intervertebral distance, kidney area (Kidney), and vastus intermedius muscle thickness (VI). Exercise and Exercise + AG values that are statistically different from the control condition are denoted by * (p < 0.05), Exercise + AG values that are statistically different from the Exercise group are denoted by ^#^ (p < 0.05).

Parameter	Control group	Exercise group	Exercise + AG group
CC IMT	6.79 ± 6.96	−6.04 ± 4.37*	−6.55 ± 4.22*
DI	−29.0 ± 16.4	0.81 ± 10.0*	33.9 ± 25.1*^#^
CC RI	−3.82 ± 5.97	−4.29 ± 4.79	−0.70 ± 3.96
Qcc	−0.11 ± 28.0	−9.71 ± 27.7	−8.83 ± 32.7
Tib RI	−33.7 ± 25.9	−42.0 ± 17.1	−30.7 ± 27.4
Aorta	−5.81 ± 11.1	14.0 ± 22.0	2.10 ± 5.57
SV	−1.52 ± 33.3	9.34 ± 32.7	20.3 ± 11.4
EF	−17.3 ± 8.34	2.38 ± 17.1*	7.50 ± 10.1*
PW	−3.94 ± 7.94	−6.18 ± 6.38	0.14 ± 9.89
JV vol	176 ± 239	42.9 ± 60.1	116 ± 129
PV	11.9 ± 14.0	4.16 ± 6.66	−3.85 ± 7.59*
C Vert	8.87 ± 15.1	11.6 ± 8.67	8.63 ± 17.8
L Vert	8.30 ± 12.7	4.40 ± 20.5	8.15 ± 12.4
Kidney	11.6 ± 15.7	14.8 ± 20.4	12.2 ± 20.3
VI	−23.1 ± 12.2	9.13 ± 18.5*	24.3 ± 17.6*

Assessments of the common carotid artery found significant HDT by group interactions for intima media thickness (Figure 2A; p < 0.001), and the distensibility index (DI) (Figure 2B; p < 0.001). In the control condition, CC IMT was increased with HDT (p = 0.002), and CC DI was decreased (p < 0.001). In contrast, CC IMT was decreased with both EX (p = 0.002) and EX-AG (p < 0.001) where CC DI was not changed with EX (p = 0.893) and increased with EX-AG (p < 0.001). Assessment of the percent changes (Table 1) confirmed that there was no difference between the EX and EX-AG groups for the CC IMT response to HDT (p = 0.98), but there was a statistically significant difference in the DI response between the EX and EX-AG groups (p = 0.004). CC RI (Figure 2C) was decreased with HDT in all groups (p = 0.007) and common carotid artery blood flow (Figure 2D) was not changed with HDT.

A significant group by HDT interaction was found for ejection fraction (Figure 3A; p < 0.001) where EF was decreased in the control group (p < 0.001) but not changed with EX (p = 0.235) or EX-AG (p = 0.208). Assessment of the percent changes in EF (Table 1) found that responses in both countermeasure groups were different from the control condition (p = 0.014 and p = 0.003 for EX and EX-AG respectively) but not different between each other (p = 0.715). Left ventricle Posterior wall thickness was decreased with HDT (Figure 3B; p = 0.045) with no effect of experimental group. Cardiac stroke volume (Table 1) and abdominal aorta diameter (Figure 3C) were not changed with HDT.

Tibial vascular resistance (Figure 3D) decreased (p < 0.001) and jugular vein volume (Figure 3E) increased (p = 0.005) in all three groups with HDT. A significant group by HDT interaction was found for portal vein diameter (Figure 3F; p = 0.019) were PV diameter increased in the control group (p = 0.006) but was not changed with EX (p = 0.235) or EX-AG (p = 0.208). Assessment of the precent changes in PV diameter (Table 1) found a difference between the control condition and EX-AG (p = 0.013), but not between EX and the control condition (p = 0.291) or EX and EX-AG (p = 0.266).

Quantitative assessments of non-cardiovascular structures found an increase in kidney long axis area (Figure 4C; p = 0.008) with HDT. Cervical intervertebral distance was also increased with HDT (Figure 4A; p = 0.005) but not affected by C1 (EX) or C2 (Ex-AG) countermeasures. Conversely lumbar intervertebral distance (Figure 4B) was not changed in any of the three groups. Vastus intermedius thickness measured on a transverse view of the thigh (Figure 4D) showed a significant group by HDT interaction effect (p > 0.001) with thickness decreasing in the control group (p < 0.001), not changing in C1 (EX p = 0.119), and increasing in C2 (EX-AG p = 0.002) after HDT. Assessment of percent change with HDT (Table 1) found that the VI response was different between EX and the control group (p = 0.002) and EX-AG and the control group (p < 0.001) but not between EX and EX-AG (p = 0.177).

## Discussion

The primary objective of this experiment was to quantify the effects of 55 days of HDT bedrest on vascular and organ structures assessed using ultrasound imaging and evaluate the efficacy of two countermeasures; aerobic exercise (EX), like that already used by astronauts in space, and exercise with artificial gravity exposure (EX-AG) which has not been tested yet during spaceflight. Past ground-based and spaceflight experiments using ultrasound assessments have identified several unexpected changes in vascular parameters including altered carotid artery wall and jugular vein properties. As these alterations were identified incidentally, it is possible that other organs and blood vessels might also be affected by spaceflight or conditions associated with spaceflight analogue studies. Therefore, the current bedrest study investigated a large number of organs and blood vessels accessible to ultrasound imaging to provide greater understanding of the effects of HDT on the entire body. Additionally, this study looked at the effects of two different countermeasure protocols on potential vascular and organ structure changes with HDT.

Only ultrasound imaging was used in the present study as it is currently the only imaging modality available during spaceflight. This allows for direct comparisons to be made between response to HDT and spaceflight to both understand similarities and differences between adaptations to both conditions and the potential benefit of various countermeasure protocols. Other imaging methods (CT Scan MRI, PQct) were used during the current bedrest study by other experimental groups and will be available in the future for comparison with ultrasound data according to a data sharing agreement.

In the present HDT study, a set of 10 organs and blood vessels were successfully investigated by ultrasound following a similar protocol to that used during a 40-day isolation experiment (Arbeille et al., 2023), dry immersion experiment (Arbeille et al., 2017b), and the program presently running onboard the International Space Station (Routine ultrasound CIPHER program). The effect of HDT was clearly identified as well as the effect of each of the two countermeasures. The main new findings of the study were that HDT resulted in adaptations that altered ultrasound measurements in more vascular and non-vascular structures than have previously been reported, and that the exercise and exercise artificial gravity had a protective effect on some of the parameters assessed. For some measures of carotid artery distensibility and portal vein diameter, exercise with artificial gravity exposure completely negated the HDT effect possibly due to artificial gravity counteracting the microgravity induced fluid shift contributing to the maintenance of vascular structure.

The common carotid artery intima media thickness (IMT) was slightly but significantly increased in the control participants consistent with the findings of increased IMT during a spaceflight (Arbeille et al., 2017a), during the simulated space exploration (Mars 500 confinement study) (Arbeille et al., 2014), and the 180 days CELSS confinement (Yuan et al., 2019). The morphological transformation of the carotid wall intima was found to be associated with alteration of the liver metabolism after a 6-month spaceflight (Hughson et al., 2016) and similar to the IMT increase observed with 20 years of aging on Earth (Hughson et al., 2016; Arbeille et al., 2017a), but the mechanisms for this change remain unclear.

In the present bedrest the increase in IMT in the control group was smaller (∼6%) than in spaceflight or long-term confinement (∼15%). This was potentially due to the duration of the study only being 55 days compared to 180 days of spaceflight (Arbeille et al., 2017a) or 520 days of confinement (Arbeille et al., 2014; Yuan et al., 2019). Additionally, the level of environmental stress could also contribute to the differences. Participants in the current study interacted daily with approximately 15 different people (nurses, doctors, scientists, manager and administrator…). In contrast, during spaceflight and confinement, study participants are limited to interacting only with the same five other crewmembers and were confined in a smaller space which could produce a more stressful environment resulting in greater vascular changes. The reduction in IMT thickness in both the C1, EX group and the C2, EX-AG group suggest that the exercise counteracts the adverse effect of HDT at the carotid level with the additional benefit making the carotid IMT thinner (younger) compared to pre HDT.

Along with the increase in carotid artery intima media thickness, the carotid distensibility (DI) was significantly decreased in the control group. This is consistent with previous observations during spaceflight and dry immersion (Hughson et al., 2016; Arbeille et al., 2017a; Greaves et al., 2021). That DI was reduced less in the C1, (EX) group and slightly increased in the C2, (EX-AG) subjects which suggests the protective effects of the exercise and an additional benefit of artificial gravity exposure. While decreased DI indicates a stiffer arterial wall, similar to aging, exercise alone and exercise with artificial gravity exposure contributed to maintain vessel wall properties or make the vessel more compliant (younger).

The carotid vascular resistance index (CC RI) and carotid artery blood flow (Qcc) were not altered with HDT in the current study. This is consistent with previous observations during spaceflight and spaceflight analog experiments (Arbeille et al., 2017b). The CC RI provides an indication of total downstream vascular resistance. Cerebral vascular resistance (measured on the middle cerebral artery) likely increased in relation to brain and fluid compressive effect on intracranial vessels (Roberts et al., 2017; Arbeille et al., 2017b; Arbeille et al., 2021b) as jugular vein volume was found to increase in all groups with HDT indicating a fluid shift towards the head similar to what has been previously observed with spaceflight and dry immersion (Arbeille et al., 2001; Arbeille et al., 2017b; Marshall-Goebel et al., 2018; Arbeille et al., 2021b; Greaves et al., 2022). However, vascular resistance outside the skull (measured on the external carotid) may have decreased with the presence of facial skin edema resulting in no change in the vascular resistance index measured at the level of the common carotid artery. Additionally, CC RI, Qcc, and JV vol were not affected by the countermeasures indicating that the exercise and artificial gravity countermeasures were either ineffective as alleviating fluid congestion in the neck and head or that the effects were transient.

With HDT, while cardiac stroke volume was maintained, the posterior wall thickness of the left ventricle tended to decrease in all three groups. In a previous study (Greaves et al., 2019) intensive physical activity maintained posterior wall thickness after 21 days of HDT. In contrast, it appears that the daily exercise performed in the current study was not sufficient to prevent changes in cardiac morphology with the longer duration HDT. However, ejection fraction was maintained in the countermeasures groups and decreased in the control group suggesting that the countermeasure did have a protective effect on cardiac function. These results are consistent with previous studies which have shown reductions in ejection fraction with HDT and spaceflight (Arbeille et al., 2001; Greaves et al., 2019) and protective effects of physical activity on the left ventricle function (Greaves et al., 2019; Arbeille et al., 2023).

Consistent with previous studies of spaceflight, bedrest, and dry immersion (Arbeille et al., 2015; Arbeille et al., 2017b) portal vein diameter was increased with HDT in the control group due to headward fluid shifts and blood pooling into the liver. In contrast, portal vein diameter was not increased in either of the countermeasure groups suggesting a protective effect. However, the countermeasures were not completely effective against the fluid shift effect as the right kidney area increased with HDT in all three groups.

The cervical intervertebral distance (C Vert) increased in all three groups with HDT. This is likely related to the −6° head down position that participants maintained throughout the study. During −6° HDT, gravity acts to pull the weight of the head away from the body, lengthening the cervical column. Other studies have measured cervical disc height before, during, and post spaceflight using ultrasound, but the correlation with MRI data was poor (Harrison et al., 2018). Regardless, ultrasounds assessment can still determine changes in this parameter even if the absolute values are not completely accurate (Greaves et al., 2024).

In contrast to the cervical vertebra, lumber intervertebral distance (L Vert) did not change in any of the three groups. This is also contrary to lumbar spinal changes expected to occur with spaceflight as many astronauts report back pain and have a greater risk of intervertebral disc herniation after spaceflight (Bailey et al., 2017; Bellavy et al., 2016). Additionally, ultrasound assessments have also identified spinal structural changes (herniation, desiccation, osteophytes) with spaceflight on the ISS (Garcia et al., 2017). In the current study, no abnormal structural changes in the spine were noted and the lack of changes in lumbar intervertebral distance could be due to gravity still being present throughout HDT preventing lengthening of the lumbar spine to the same extent as exposure to microgravity during spaceflight.

At the leg level, tibial vascular resistance (Tib RI) was significantly decreased in all three groups likely due to the emptying of fluid from the legs and the absence of muscular activity. The measures of vastus intermedius thigh muscle thickness (VI) confirmed the atrophy of this muscle with HDT in the control group. As the vastus intermedius contributes to maintaining upright posture in normal gravity, similar atrophy may also be present in other postural leg muscles. In contrast to the control condition, while the countermeasures did not affect tibial vascular resistance, they did maintain VI muscle thickness in the C1, EX group and increased thickness in the C2, EX-AG group.

The systematic investigation, of 10 organs and blood vessels accessible to ultrasound in the present study showed that many systems are affected with prolonged HDT bedrest. Many of these adaptations appear to be related to inactivity and fluid shifts as the exercise and artificial gravity countermeasures were able to provide some protective effects. Additionally, a similar investigation performed during 40-day of isolation in a cavern (Arbeille et al., 2023) where participants remained active in a large habitat and did not experience fluid shifts, showed no changes at all in organs and blood vessels investigated.

Currently, the same ultrasound protocol used in this study is running onboard ISS (CIPHER Routine ultrasound program) using remote ultrasound imaging, teleoperated from the ground, to assist astronauts with minimal ultrasound experience, in obtaining images that can be used for diagnostics to determine the effects of long duration spaceflight and investigate the recovery of these parameters as astronauts return to Earth (Arbeille et al., 2023). To date, none of the changes observed, with the exception of one reported case of jugular vein thrombosis during flight (Marshall-Goebel et al., 2019), have related to pathological conditions, and the corresponding parameters recover 6 months after returning to Earth. Preliminary results from another study (VASC AGING), have indicated that vascular parameters, affected by 6 months of spaceflight, may generally recover 6 month after returning to Earth suggesting the transient nature of the observed adaptations (personal data). However, with longer duration spaceflight or HDT, it is still unknown if these parameters will recover in a similar timeline or if the observed changes will become the starting point of pathological situations such as vessel atheromatous process, vein thrombosis, organ cellular/structural change, and subsequent metabolic disorders.

## Conclusion

After 55 days of continuous −6° head down bedrest, changes in ultrasound measures of cardiovascular targets were similar to those previously observed during long duration spaceflight. Additionally, the ultrasound evaluation identified HDT induced changes on non cardiovascular targets like the leg muscle atrophy, increased cervical intervertebral distance and kidney area. The exercise (EX) and artificial gravity (EX-AG) countermeasures prevented changes in some of the measured variables, but not all. Results from this study help to provide a framework for interpreting ultrasound diagnostic assessments during long duration spaceflight.

## New and noteworthy

Results from this study indicate significant physiological effects of 55-day bedrest in the control subjects while the exercise alone and the exercise with artificial gravity countermeasures and maintained the parameters relatively stable.

## Data Availability

The raw data supporting the conclusions of this article will be made available by the authors, without undue reservation.

## References

[B1] Arbeille P. Avan P. Treffel L. Zuj K. Normand H. Denise P. (2017b). Denise Jugular and Portal Vein Volume, Middle Cerebral Vein Velocity, and Intracranial Pressure in Dry Immersion. Aerosp. Med. Hum. Perform. 88, 457, 462. 10.3357/AMHP.4762.2017 28417833

[B2] Arbeille P. Chaput D. Zuj K. Depriester A. Maillet A. Belbis O. (2018). Remote echography between a ground control center and the international space station using a tele-operated echograph with motorized probe. Ultrasound Med. Biol. 44, 2406–2412. 10.1016/j.ultrasmedbio.2018.06.012 30093338

[B3] Arbeille P. Fomina G. Roumy J. Alferova I. Tobal N. Herault S. (2001). Adaptation of the left heart, cerebral and femoral arteries, and jugular and femoral veins during short- and long-term head-down tilt and spaceflights. Eur. J. Physiol. 86, 157–168. 10.1007/s004210100473 11822475

[B4] Arbeille P. Greaves D. Chaput D. Maillet A. Hughson R. L. (2021a). Index of reflectivity of ultrasound radio frequency signal from the carotid artery wall increases in astronauts after a 6 mo spaceflight. Ultrasound Med. Biol. 47, 2213–2219. 10.1016/j.ultrasmedbio.2021.03.028 34001406

[B5] Arbeille P. Provost R. Vincent N. Aubert A. (2014). Adaptation of the main peripheral artery and vein to long term confinement (MARS 500). PLoS One 9, e83063. 10.1371/journal.pone.0083063 24475025 PMC3903485

[B6] Arbeille P. Provost R. Zuj K. (2017a). Carotid and femoral arterial wall distensibility during long-duration spaceflight. Aerosp. Med. Hum. Perform. 88, 924–930. 10.3357/AMHP.4884.2017 28923141

[B7] Arbeille P. Provost R. Zuj K. Vincent N. (2015). Measurements of jugular, portal, femoral, and calf vein cross-sectional area for the assessment of venous blood redistribution with long duration spaceflight (Vessel Imaging Experiment). Eur. J. Appl. Physiol. 115, 2099–2106. 10.1007/s00421-015-3189-6 25991027

[B8] Arbeille P. Zuj K. Besnard S. Mauvieux B. Hingrand C. Delaunay P.-L. (2023). Ultrasound assessments of organs and blood vessels before and after 40 days isolation in a cavern (Deep Time experiment 2021). Front. Physiol. 14, 1174565. 10.3389/fphys.2023.1174565 37168224 PMC10164955

[B9] Arbeille P. Zuj K. A. Macias B. R. Ebert D. J. Laurie S. S. Sargsyan A. E. (2021b). Lower body negative pressure reduces jugular and portal vein volumes and counteracts the elevation of middle cerebral vein velocity during long-duration spaceflight. J. Appl. Physiol. 131, 1080–1087. 10.1152/japplphysiol.00231.2021 34323592 PMC8461809

[B10] Bailey J. F. Miller S. L. Khieu K. O'Neill C. W. Healey R. M. Coughlin D. G. (2017). From the international space station to the clinic: how prolonged unloading may disrupt lumbar spine stability. Spine J. 18 (17). 7–14. 10.1016/j.spinee.2017.08.261 [Epub ahead of print] PubMed PMID: 28962911 . 28962911 PMC6339989

[B11] Belavy D. L. Adams M. Brisby H. Cagnie B. Danneels L. Fairbank J. (2016). Disc herniations in astronauts: what causes them, and what does it tell us about herniation on earth? Eur. Spine J. 25, 144–154. 10.1007/s00586-015-3917-y 25893331

[B12] Garcia K. M Harrison M. F. Sargsyan A. E. Douglas P. D. Scott A. (2017). Real-timeUltrasound assessment of astronaut spinal anatomy and disorders on the international space statio. J. UltrasoundMed 37, 987–999. 10.1002/jum.14438 28960477

[B13] Greaves D. Arbeille P. Guillon L. Zuj K. Caiani E. G. (2019). Effects of exercise countermeasure on myocardial contractility measured by 4D speckle tracking during a 21-day head-down bed rest. Eur. J. Appl. Physiol. 119, 2477–2486. [Epub Ahead Of Print]. 10.1007/S00421-019-04228-0 31531733

[B14] Greaves D. Guillon L. Besnard S. Navasiolava N. Arbeille P. (2021). 4 day in dry immersion reproduces partially the aging effect on the arteries as observed during 6 month spaceflight or confinement. Npj microgravity, Springer 7, 43. 10.1038/s41526-021-00172-6 34728651 PMC8564509

[B15] Greaves D. Petersen L. Arbeille P. (2024) “Cervical intervertebral distance measured using 3D ultrasound after dry immersion and 6 Month spaceflight,” in Proceeding HRP meeting galveston.

[B16] Harrison M. F. Garcia K. M. Sargsyan A. E. Ebert D. Riascos-Castaneda R. F. Dulchavsky S. A. (2018). Preflight, in-flight, and postflight imaging of the cervical and lumbar spine in astronauts. Aerosp. Med. Hum. Perform. 89 (1), 32–40. 10.3357/AMHP.4878.2018 29233242

[B17] Hughson R. L. Robertson A. D. Arbeille P. Shoemaker J. K. Rush J. W. E. Fraser K. S. (2016). Increased postflight carotid artery stiffness and inflight insulin resistance resulting from 6-mo spaceflight in male and female astronauts. Am. J. Physiol. - Hear Circ. Physiol. 310, H628–H638. 10.1152/ajpheart.00802.2015 26747504

[B18] Marshall-Goebel K. Laurie S. S. Alferova I. V. Arbeille P. Auñón-Chancellor S. M. Ebert D. J. (2019). Assessment of jugular venous blood flow stasis and thrombosis during spaceflight. JAMA Netw. open 2, e1915011. 10.1001/jamanetworkopen.2019.15011 31722025 PMC6902784

[B19] Marshall-Goebel K. Stevens B. Rao C. V. Suarez J. I. Calvillo E. Arbeille P. (2018). Internal jugular vein volume during head-down tilt and carbon dioxide exposure in the SPACECOT study. Aerosp. Med. Hum. Perform. 89 (4), 351–356. 10.3357/AMHP.4934.2018 29562964

[B20] Patterson C. A. Greaves D. K. Robertson A. Hughson R. Arbeille P. (2023). Motorized 3D ultrasound and jugular vein dimension measurement on the international space station. Aerosp. Med. Hum. Perform. 94, 466–469. 10.3357/amhp.6219.2023 37194183

[B21] Roberts D. R. Albrecht M. H. Collins H. R. Asemani D. Chatterjee A. R. Spampinato M. V. (2017). Effects of spaceflight on astronaut brain structure as indicated on MRI. N. Engl. J. Med. 377, 1746–1753. 10.1056/nejmoa1705129 29091569

[B22] Yuan M. Custaud M. C. Xu Z. Wang J. Yuan M. Tafforin C. (2019). Multi-system adaptation to confinement during the 180-day controlled ecological life support system (CELSS) experiment. Front. Physiology 10, 575. 10.3389/fphys.2019.00575 PMC6536695 31164833

